# Dehydrocostus lactone attenuates osteoclastogenesis and osteoclast‐induced bone loss by modulating NF‐κB signalling pathway

**DOI:** 10.1111/jcmm.14492

**Published:** 2019-06-21

**Authors:** Bin Hu, Fengfeng Wu, Zhongli Shi, Bin He, Xiang Zhao, Haobo Wu, Shigui Yan

**Affiliations:** ^1^ Department of Orthopedic Surgery, the Second Affiliated Hospital Zhejiang University School of Medicine Hangzhou China; ^2^ Orthopedic Research Institute of Zhejiang University Hangzhou China; ^3^ Department of Orthopedic Surgery, Huzhou Central Hospital Zhejiang University Huzhou Hospital Huzhou China

**Keywords:** dehydrocostus lactone, NFATc1, osteoclast, RANKL

## Abstract

Osteolysis is characterized by overactivated osteoclast formation and potent bone resorption. It is enhanced in many osteoclast‐related diseases including osteoporosis and periprosthetic osteolysis. The shortage of effective treatments for these pathological processes emphasizes the importance of screening and identifying potential regimens that could attenuate the formation and function of osteoclasts. Dehydrocostus lactone (DHE) is a natural sesquiterpene lactone containing anti‐inflammatory properties. Here, we showed that DHE suppressed receptor activator of nuclear factor‐κB ligand (RANKL)‐induced osteoclast formation and osteoclast marker gene expression. It also inhibited F‐actin ring formation and bone resorption in a dose‐dependent manner in vitro. Moreover, DHE inhibited the RANKL‐induced phosphorylation of NF‐κB, mitigated bone erosion in vivo in lipopolysaccharide‐induced inflammatory bone loss model and particle‐induced calvarial osteolysis model. Together, these results suggest that DHE reduces osteoclast‐related bone loss via the modulation of NF‐κB activation during osteoclastogenesis indicating that it might be a useful treatment for osteoclast‐related skeletal disorders.

## 1 INTRODUCTION

Bone remodelling is a delicate homeostasis involving the regulation of both osteoblast and osteoclast activities. Maintenance of this balance helps prevent disorders such as rheumatoid arthritis, osteoporosis, and multiple myeloma‐related bone loss.^1^ In clinical practice, inflammatory bone loss remains difficult to treat, especially in complicated orthopedic trauma and arthroplasty cases. Inflammation of the skeletal system activates immune reactions and produces cytokines involved in osteoclast recruitment and differentiation, leading to excessive bone resorption.^2^ However, an effective regimen for the treatment of inflammatory bone loss is lacking.

Osteoclasts are multinucleate, tartrate‐resistant acid phosphatase (TRAP)‐positive cells originating from the monocyte‐macrophage lineage. They play a crucial role in both physiological bone remodelling and pathological bone loss for the unique process of bone resorption. Osteoclastogenesis is induced by receptor activator of nuclear factor‐κB ligand (RANKL) in the presence of macrophage colony‐stimulating factor (M‐CSF).^3^ Receptor activator of nuclear factor‐κB ligand belongs to the tumour necrosis factor (TNF) superfamily; binding with its receptor, RANK, on the surface of osteoclast precursors recruits adaptor TNF receptor‐associated factors (TRAFs). This process is important for osteoclast formation because it activates several downstream signalling pathways and the transcription factor activator protein 1 (AP‐1), leading to full activation of T‐cell cytoplasmic 1 (NFATc1).^4^ The expression of NFATc1 modulates osteoclast differentiation and functions via the induction of osteoclast‐specific genes, including those encoding dendritic cell‐specific transmembrane protein (DC‐STAMP), TRAP, cathepsin K, calcitonin receptor (CTR), and matrix metalloproteinase 9 (MMP‐9).^5^, ^6^

Natural products have been an increasingly important source for drug development.^7^ We recently identified dehydrocostus lactone (DHE) as an effective inhibitor of osteoclastogenesis and osteoclast‐related bone loss. Dehydrocostus lactone is a natural sesquiterpene lactone derived from the roots of *Saussurea lappa*, and it has been reported to have anti‐inflammatory, anti‐ulcer, anti‐tumour, and immunomodulatory properties.^8^, ^9^ However, the effect of DHE on osteoclast formation has not been reported. It has been shown that proinflammatory cytokines promote osteoclastogenesis,^10^ and overactivation of osteoclast activity plays an essential part in the origin of bone homeostatic imbalances.^11^ Thus, osteoclasts could be a key target in the treatment of inflammatory bone loss. Lipopolysaccharide (LPS) and titanium particles are both potent inducers of the immune system, and they play critical roles in the development of osteolytic bone loss.^12^ The purpose of this study was therefore to investigate the effects of DHE on osteoclastogenesis and on murine models of LPS‐induced bone loss and particle‐induced calvarial osteolysis, and to characterize the underlying mechanism of these processes.

## 2 MATERIALS AND METHODS

### 2.1 Reagents and antibodies

Foetal bovine serum and Minimum Essential Medium Eagle Alpha Modification (α‐MEM) were obtained from Gibco (Sydney, Australia). Dehydrocostus lactone was purchased from Selleck (Shanghai, China), and Cell Counting Kit‐8 (CCK‐8) was obtained from Dojindo Molecular Technologies (Shanghai, China). Recombinant murine RANKL and M‐CSF were purchased from R&D Systems (Minneapolis, MN). Primary antibodies against extracellular signal‐regulated kinase (ERK), p‐ERK, c‐Jun N‐terminal kinase (JNK), p‐JNK, p38, p‐p38, p65, p‐p65, IκBα, p‐IκBα, IκB kinase β (IKKβ), p‐IKKα/β, NFATc1/NFAT2, c‐Src, and β‐actin were purchased from Cell Signaling Technology (Danvers, MA). Antibodies against cathepsin K were obtained from Abcam (Cambridge, MA). Rhodamine‐conjugated phalloidin was from Cytoskeleton (Denver, CO). Titanium particles were purchased from Alfa Aesar (#00681; Alfa Aesar, Ward Hill, MA). The TRAP staining kit, Tris, glycine, dimethylsulfoxide (DMSO), and sodium dodecyl sulfate were from Sigma‐Aldrich (St. Louis, MO) unless otherwise stated.

### 2.2 Cell culture and osteoclast differentiation

Primary murine bone monocyte/macrophage precursors were isolated from the unfractionated long bones of 6‐week‐old C57BL/6 mice and differentiated into bone marrow‐derived macrophages (BMMs) in α‐MEM containing 30 ng/mL of M‐CSF for 3‐4 days. The cells were cultured in at 37°C in 5% CO_2_ incubator, and the medium was changed every 2 days. The effect of DHE on BMM viability was determined using a CCK‐8 assay. Cells were plated into 96‐well culture plates at 8 × 10^3^/well for 1 day, and then incubated with different concentrations of DHE for 4 days. The cells were then incubated for 4 hours after the addition of 10 μL of CCK‐8 buffer to each well, and the optical density (OD) at 450 nm was measured on an ELX808 absorbance microplate reader (BioTek, Winooski, VT). During osteoclast differentiation, BMMs were seeded (8 × 10^3^/well) into 96‐well plates and cultured with osteoclastogenic medium (complete α‐MEM containing 30 ng/mL of M‐CSF and 100 ng/mL of RANKL) and incubated with the indicated concentrations of DHE for 5 days. The cells were then fixed with 4% paraformaldehyde and stained using a TRAP staining kit according to the manufacturer's instructions. Tartrate‐resistant acid phosphatase‐positive cells containing ≥3 nuclei were considered mature osteoclasts. The number and spread area of mature osteoclasts were quantified using Imagej (National Institutes of Health [NIH], Bethesda, MD).

### 2.3 Immunofluorescence staining

The effect of DHE on F‐actin ring formation was visualized using rhodamine‐phalloidin staining. Bone marrow‐derived macrophages were plated into 96‐well plates and treated with the indicated concentrations of DHE in the presence of 30 ng/mL of M‐CSF and 100 ng/mL of RANKL for 5 days. The cells were then fixed in 4% paraformaldehyde at room temperature; and permeabilized with 0.1% Triton X‐100 in phosphate‐buffered saline. F‐actin was stained with rhodamine‐phalloidin in 2% bovine serum albumin for 1 hour. The nuclei were stained with 4′,6‐diamidino‐2‐phenylindole for 5 minutes. Fluorescence images were acquired using a Leica DMI4000B fluorescence microscope (Wetzlar, Germany). Three fields were randomly selected, and the numbers and sizes of the F‐actin rings were calculated using Imagej (NIH).

### 2.4 Bone resorption pit assay

The effects of DHE on osteolytic bone resorption in vitro were assessed on bone discs. An equal number of BMM‐derived pre‐osteoclasts were seeded onto bone discs and incubated overnight. The cells were then treated with or without 4 μmol/L DHE in osteoclastogenic medium for 2‐3 days. The discs were fixed with 4% paraformaldehyde, and adhered cells were removed by gentle brushing. Three random views in each disc were visualized using a Hitachi S‐3700N Scanning Electron Microscopy (Tokyo, Japan), and the resorbed areas were quantified by Imagej (NIH).

### 2.5 Quantitative polymerase chain reaction

Bone marrow‐derived macrophages were plated into six‐well culture plates at 3 × 10^4^/well and cultured in osteoclastogenic medium with serial doses of DHE (0, 0.5, 1, 2, and 4 μmol/L) for 5 days. Total cellular RNA was isolated using RNAiso Reagent (TaKaRa, Dalian, China) and quantified using a NanoDrop 2000 (Thermo Fisher Scientific, Waltham, MA). Total RNA was reverse‐transcribed into cDNA according to the manufacturer's instructions. Quantitative polymerase chain reaction (PCR) was performed in triplicate using 2 μL of cDNA as the template and SYBR^®^ Premix Ex TaqTM II (TaKaRa) with an ABI StepOnePlus system (Applied Biosystems, Warrington, UK). All tests were run in triplicate, and β‐actin was used as the housekeeping gene. Gene expression was analysed using the 2^−∆∆Ct^ method. The murine primer sequences were as follows: NFATc1, forward 5′‐CCGTTGCTTCCAGAAAATAACA‐3′ and reverse 5′‐TGTGGGATGTGAACTCGGAA‐3′, DC‐STAMP, forward 5′‐AAAACCCTTGGGCTGTTCTT‐3′ and reverse 5′‐AATCATGGACGACTCCTTGG‐3′, cathepsin K, forward 5′‐CTTCCAATACGTGCAGCAGA‐3′ and reverse 5′‐TCTTCAGGGCTTTCTCGTTC‐3′, CTR, forward 5′‐TGCTGGCTGAGTGCAGAAACC‐3′ and reverse 5′‐GGCCTTCACAGCCTTCAGGTAC‐3′, TRAP, forward 5′‐CACTCCCACCCTGAGATTTGT‐3′ and reverse 5′‐CCCCAGAGACATGATGAAGTCA‐3′, MMP‐9, forward 5′‐CAAAGACCTGAAAACCTCCAA‐3′ and reverse 5′‐GGTACAAGTATGCCTCTGCCA‐3′, V‐ATPase a3, forward 5′‐GCCTCAGGGGAAGGCCAGATCG‐3′ and reverse 5′‐GGCCACCTCTTCACTCCGGAA‐3′, OSCAR, forward 5′‐CCTAGCCTCATACCCCCAG‐3′ and reverse 5′‐CGTTGATCCCAGGAGTCACAA‐3′, β‐actin, forward 5′‐TCTGCTGGAAGGTGGACAGT‐3′ and reverse 5′‐CCTCTATGCCAACACAGTGC‐3′.

### 2.6 Western blot analysis

To investigate the effects of DHE on RANKL‐induced signalling pathways, RAW264.7 cells were seeded into six‐well plates at 8 × 10^5^/well in complete α‐MEM and incubated overnight. Cells were then pretreated with or without 4 μmol/L DHE for 4 hours before being stimulated by 50 ng/mL of RANKL for 0, 5, 15, 30, or 60 minutes. To determine the effects of DHE on osteoclast‐related protein expression, BMMs were seeded into six‐well plates at 24 × 10^4^/well and cultured in complete α‐MEM with 30 ng/mL of M‐CSF and 100 ng/mL of RANKL containing DMSO or 4 μmol/L DHE for 0, 1, 3, or 5 days. Total cellular proteins were extracted using Radio Immunoprecipitation Assay lysis buffer supplemented with protease and phosphatase inhibitor cocktails (Fude Biological Technology, Hangzhou, China) for 20 minutes at 4°C. Lysates were then cleared by centrifugation at 18 400 *g* for 15 minutes at 4°C, and the supernatants were collected. Equal amounts of total protein (~30 μg/lane) were resolved using 10% SDS‐PAGE and electroblotted onto polyvinylidene difluoride membranes (Millipore, Shanghai, China). The membranes were blocked with 5% (w/v) nonfat milk in 0.1% Tris‐buffered saline and Tween 20 for 1 hour at room temperature, and then incubated with primary antibodies at 4°C overnight with gentle shaking. After washing and incubation with specific secondary antibodies for 1 hour at room temperature, the bands were detected using Enhanced Chemiluminescent Detection Reagent (Fude Biological Technology) with the Bio‐Rad XRS Chemiluminescence Detection System (Hercules, CA, USA).

### 2.7 Murine model of LPS‐induced bone loss and particle‐induced calvarial osteolysis

This study was carried out in strict accordance with the protocols of the Animal Care and Use Committee of the Second Affiliated Hospital of Zhejiang University School of Medicine. Eight‐week‐old male C57BL/6 mice were provided by Shanghai SLAC Laboratory Animal Center (Shanghai, China). For murine model of LPS‐induced bone loss, mice were randomized into four groups, with five animals in each group: DMSO‐treated (control group), LPS (5 μg/g)‐treated (LPS group), LPS + DHE (7.5 μg/g)‐treated (LPS + low DHE group) and LPS + DHE (15 μg/g)‐treated (LPS + high DHE group). Lipopolysaccharide was administered intraperitoneally on days 1 and 5. Dehydrocostus lactone was given intraperitoneally 1 day before the injection of LPS, and subsequently on every other day for up to 8 days. All mice were sacrificed by cervical dislocation, and the tibias were scanned by micro‐computed tomography (CT) (μCT 100; Scanco Medical, Bassersdorf, Switzerland). Data on bone volume/tissue volume (BV/TV), trabecular thickness (Tb.Th), trabecular number (Tb.N), and trabecular separation (Tb.Sp) were collected to evaluate the trabecular microarchitecture of the tibias. Titanium particles (#00681; Alfa Aesar) were sterilized prior to use and resuspended with PBS. For model of particle‐induced calvarial osteolysis, mice were randomized into four groups with five in each: PBS‐treated (control group), Particle‐treated (Particle group), Particle + DHE (7.5 μg/g)‐treated (Particle + low DHE group), and Particle + DHE (15 μg/g)‐treated (Particle + high DHE group). Anaesthesia was reached with intraperitoneal sodium pentobarbital (50 mg/kg), and then 30 mg of titanium particles in PBS were injected under the periosteum of calvaria in Particle group and DHE treatment groups, while equal volume of PBS was injected in control group. DHE was given intraperitoneally 1 day before the implantation of titanium particles, and subsequently on every other day for up to 2 weeks. Then calvaria were harvested for analysis of resorption area. All specimens were fixed in 4% paraformaldehyde and then decalcified in 10% EDTA (pH ~ 7.4) for 4 weeks. After decalcification, the specimens were embedded and cut into sections (3 μm thick) and then stained with haematoxylin and eosin.

### 2.8 Statistical analysis

Data are expressed as the mean ± SD. All experiments were independently carried out at least three times. Unpaired Student's *t* tests were used to compare two groups, and one‐way ANOVA tests were used for ≥three groups, with SPSS for Windows, version 19.0 (IBM Crop., Armonk, NY). A value of *P* < 0.05 indicated a significant difference between groups.

## 3 RESULTS

### 3.1 DHE inhibited the differentiation of BMMs into osteoclasts without cytotoxicity

**Figure 1:**
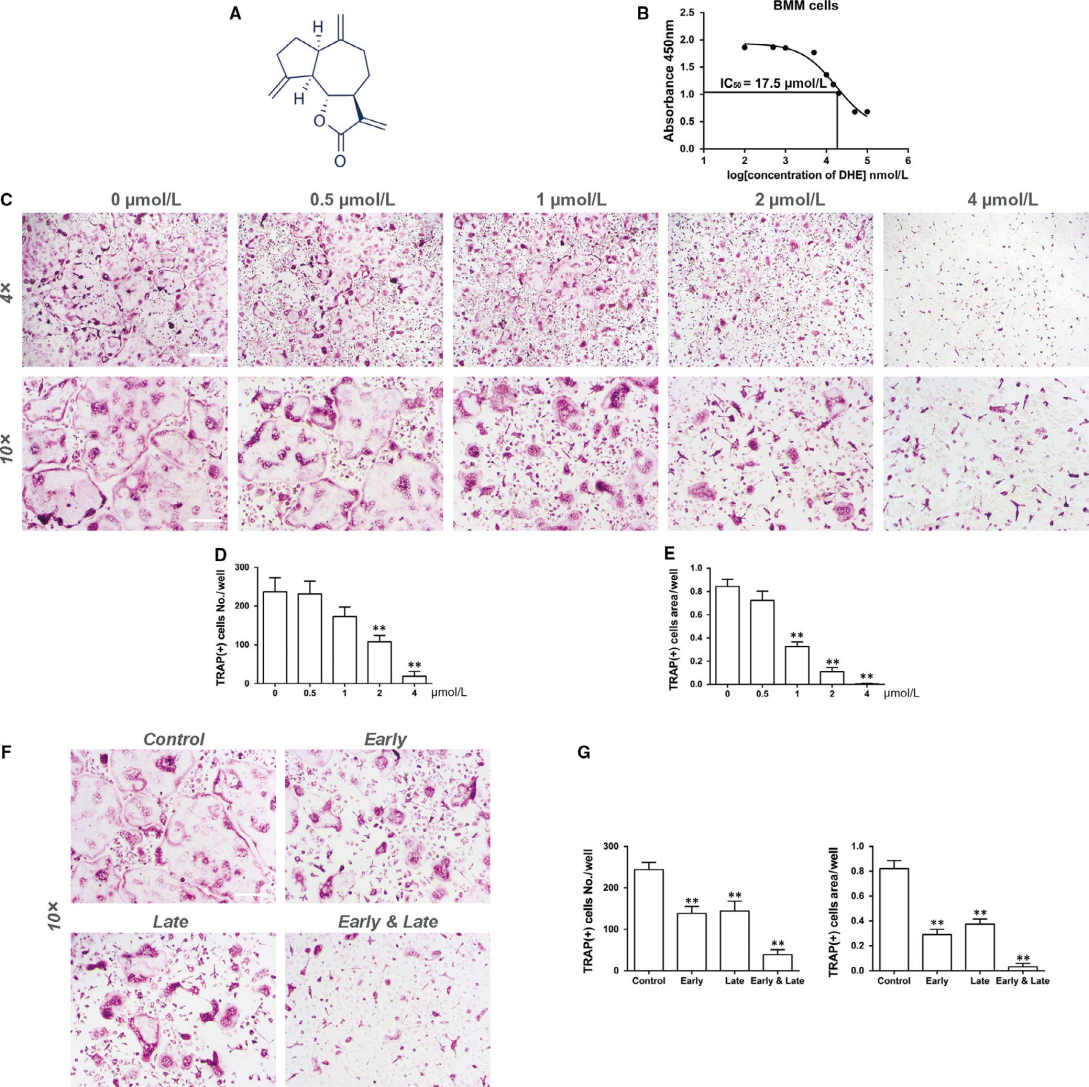
Dehydrocostus lactone (DHE) suppressed receptor activator of nuclear factor‐κB ligand (RANKL)‐induced osteoclast formation in vitro. A, The chemical structure of DHE. B, Proliferation and survival curve for bone marrow‐derived macrophages (BMMs) exposed to a range of doses of DHE for 72 h. (C‐E) BMMs were cultured with various concentrations of DHE (0, 0.5, 1, 2, or 4 μmol/L) in the presence of macrophage colony‐stimulating factor (M‐CSF, 30 ng/mL) and RANKL (100 ng/mL) for 5 d, fixed and stained with tartrate‐resistant acid phosphatase (TRAP) to visualize mature osteoclasts and measure their numbers and spread area. (F, G) BMMs were cultured with 4 μmol/L DHE on days 1‐3 (early), 3‐5 (late) or 1‐5 (early and late) in the presence of M‐CSF (30 ng/mL) and RANKL (100 ng/mL). Data were expressed as means ± SD. **P < 0.01 versus the control group. Scale bar = 500 μm for 4× images and 200 for 10× images

The cytotoxicity of DHE (Figure 1A) toward osteoclast precursor cells was determined by the CCK‐8 assay with a range of doses of DHE, and the results indicate that the IC_50_ of DHE for BMMs was 17.5 μmol/L (Figure 1B). To investigate the effect of DHE on osteoclast formation, BMMs were cultured in α‐MEM with 25 ng/mL of M‐CSF, 100 ng/mL of RANKL, and serial concentrations of DHE. The BMMs in the control wells differentiated into characteristic multinucleate TRAP‐positive cells. However, those exposed to DHE exhibited a decreased number of TRAP‐positive cells in a dose‐dependent fashion (Figure 1C‐E). The formation of multinucleate osteoclasts was almost completely inhibited at 4 μmol/L DHE, while cell viability was not affected using the same concentration. To determine which stage of osteoclastogenesis was mainly affected by DHE, BMMs were treated with 4 μmol/L DHE at the early (1‐3 days), late (3‐5 days) and early and late stages (1‐5 days) of osteoclast differentiation, respectively. DHE remarkably reduced the number and size of osteoclasts at both the early and late stage of osteoclastogenesis (Figure 1F,G). Together, these results showed that DHE inhibited osteoclast differentiation without cytotoxicity.

### 3.2 DHE suppressed RANKL‐induced osteoclast marker gene expression

**Figure 2:**
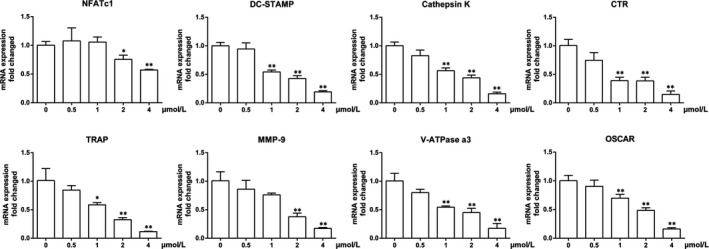
Dehydrocostus lactone (DHE) suppressed the mRNA expression of the osteoclast marker genes including NFATc1, dendritic cell‐specific transmembrane protein (DC‐STAMP), cathepsin K, calcitonin receptor (CTR), tartrate‐resistant acid phosphatase (TRAP), matrix metalloproteinase 9 (MMP‐9), c‐Fos, V‐ATPase a3 and osteoclast‐associated immunoglobulin‐like receptor (OSCAR). Bone marrow‐derived macrophages in the presence of macrophage colony‐stimulating factor (30 ng/mL) and receptor activator of nuclear factor‐κB ligand (100 ng/mL) were treated with increasing concentrations of DHE (0, 0.5, 1, 2 or 4 μmol/L) for 5 d. And mRNA expression of osteoclast marker genes was determined by quantitative polymerase chain reaction. The data were expressed as means ± SD. *P < 0.05, **P < 0.01 versus the control group

The anti‐osteoclastogenic properties of DHE on the expression of transcription factors and osteoclast‐specific markers (NFATc1, DC‐STAMP, cathepsin K, CTR, TRAP, MMP‐9, V‐ATPase a3 and osteoclast‐associated immunoglobulin‐like receptor/OSCAR) were confirmed by quantitative PCR. Compared with the control group, the mRNA expression of RANKL‐induced osteoclast marker genes was strongly suppressed by DHE in a dose‐dependent manner (Figure 2). The key transcription factor NFATc1 was decreased nearly by half in the presence of a high concentration (4 μmol/L) of DHE. Collectively, these data confirmed that DHE attenuated osteoclast formation and related gene expression during the differentiation of BMMs into osteoclasts.

### 3.3 DHE interfered with F‐actin ring formation and bone resorption in vitro

**Figure 3:**
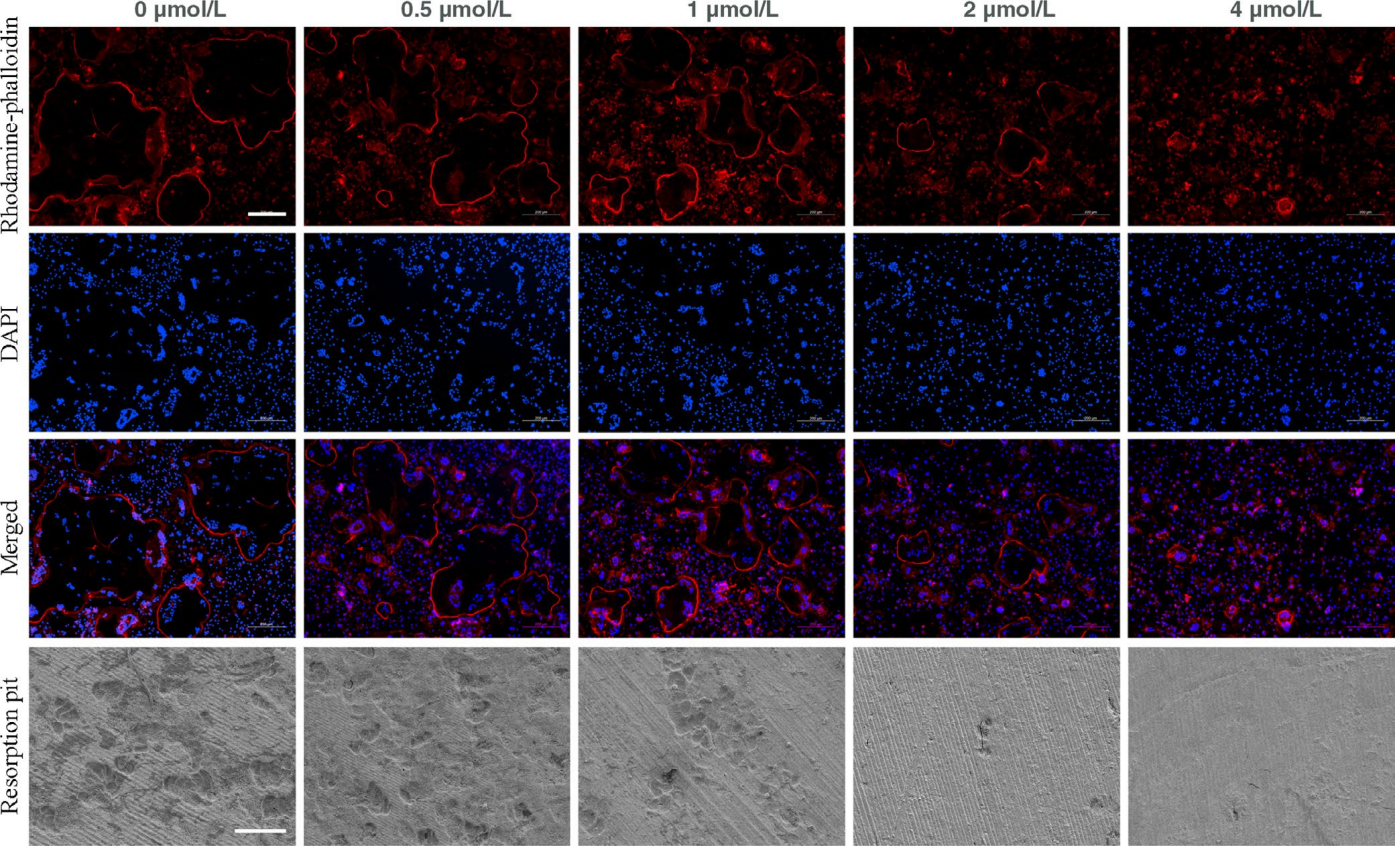
Dehydrocostus lactone (DHE) impaired F‐actin ring formation and bone resorption in vitro. Bone marrow‐derived macrophage (BMM) cells were cultured in the presence of macrophage colony‐stimulating factor (M‐CSF, 30 ng/mL) and receptor activator of nuclear factor‐κB ligand (100 ng/mL) for 5 d with serial concentrations of DHE (0, 0.5, 1, 2 or 4 μmol/L). Cells were then fixed, stained, and visualized by fluorescence microscopy. Scale bar = 200 μm for fluorescence images. For resorption pit assay, the pre‐osteoclasts were incubated on bone discs for 2‐3 d with or without 4 μmol/L DHE. Scale bar = 100 μm for SEM images

The formation of F‐actin rings is a prerequisite to the adhesion of osteoclasts to bone. To determine whether DHE impaired the formation of F‐actin rings, BMMs were seeded into 24‐well plates and cultured with 25 ng/mL of M‐CSF, 100 ng/mL of RANKL and serial concentrations of DHE. Typical F‐actin rings formed during osteoclast differentiation in the control wells, whereas treatment of osteoclasts with DHE led to a significant morphological reduction in F‐actin rings in a dose‐dependent manner (Figure 3). Number of resorption pit decreased significantly when DHE concentration reached 1 μmol/L and pit was barely seen when treated by 4 μmol/L DHE (Figure 3). This was consistent with morphologic retraction observed in F‐actin ring formation assay, which indicated that antiresorptive effects of DHE could be partially attributed to the impairment of cytoskeleton.

### 3.4 DHE attenuated RANKL‐induced NF‐κB signalling activation

**Figure 4:**
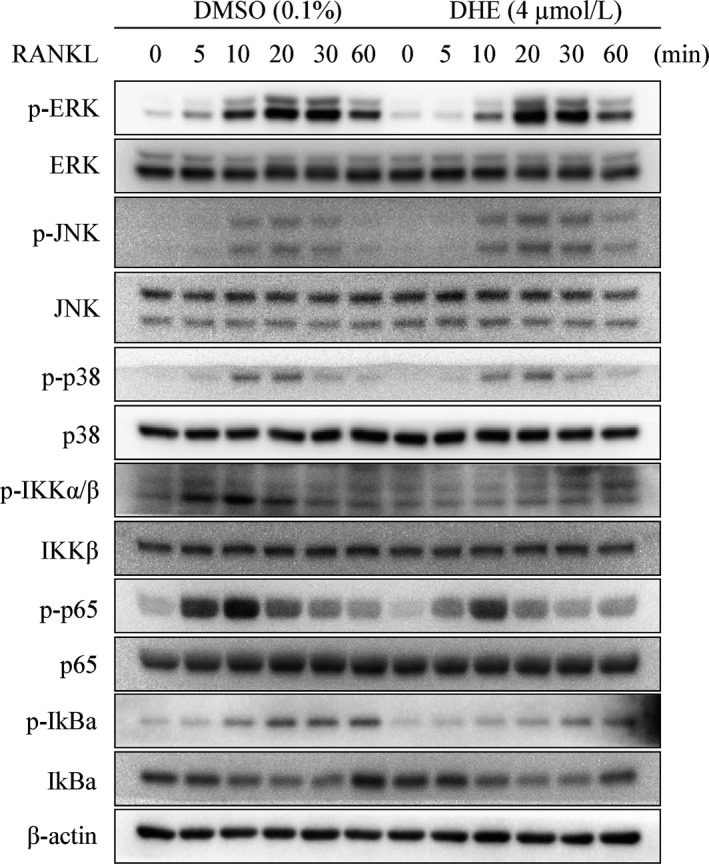
Dehydrocostus lactone (DHE) attenuated osteoclastogenesis by inhibiting the NF‐κB signalling. RAW264.7 cells were pre‐treated with dimethylsulfoxide or 4 μmol/L DHE for 4 h and then stimulated with receptor activator of nuclear factor‐κB ligand (RANKL, 50 ng/mL) for 0, 5, 15, 30 or 60 min. Total cell lysates were analysed by immune blotting using the indicated antibodies. The phosphorylation levels of extracellular signal‐regulated kinase, c‐Jun N‐terminal kinase, p38, IKKα/β, p65 and IκBa were determined after treatment with or without DHE

The downstream pathways of RANK are important for osteoclast differentiation, so we determined whether DHE modulated the phosphorylation of MAPK and NF‐κB after RANKL stimulation. The phosphorylation peaked within 60 minutes after RANKL stimulation (Figure 4). The phosphorylation of MAPK pathway was unaffected. RANKL‐induced NF‐κB activation is also crucial for osteoclast formation. As shown in Figure, activation of the upstream IKK complex, p65, and IκBα was suppressed by DHE. Taken together, these results suggest that DHE inhibited osteoclast formation by modulating the RANKL‐induced activation of NF‐κB signalling.

### 3.5 DHE impaired NFATc1 induction

**Figure 5:**
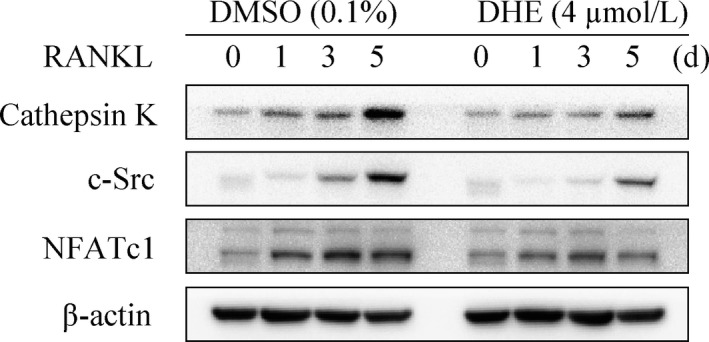
Dehydrocostus lactone (DHE) attenuated osteoclastogenesis‐related protein expression during osteoclastogenesis. Bone marrow‐derived macrophages were cultured with or without 4 μmol/L DHE in the presence of M‐CSF (30 ng/mL) and RANKL (100 ng/mL) for 0, 1, 3 or 5 d, and the expression of cathepsin K, c‐Src and NFATc1 was determined by immune blotting

NFATc1 is an expression inducer of osteoclast marker genes, and a master transcription factor during osteoclast formation.^13^ To determine whether DHE attenuated the expression of NFATc1, we investigated protein expression in the presence of DHE during osteoclastogenesis of BMMs. Figure 5 shows that the expression of NFATc1 reached a peak at day 3, while its expression was significantly down‐regulated in the presence of DHE. In addition, it attenuated the expression of cathepsin K and c‐Src in BMMs during osteoclastogenesis (Figure 5), which is critical for osteoclast formation and function.

### 3.6 The LPS‐induced bone volume loss and particle‐induced calvarial osteolysis were partially rescued by DHE

**Figure 6:**
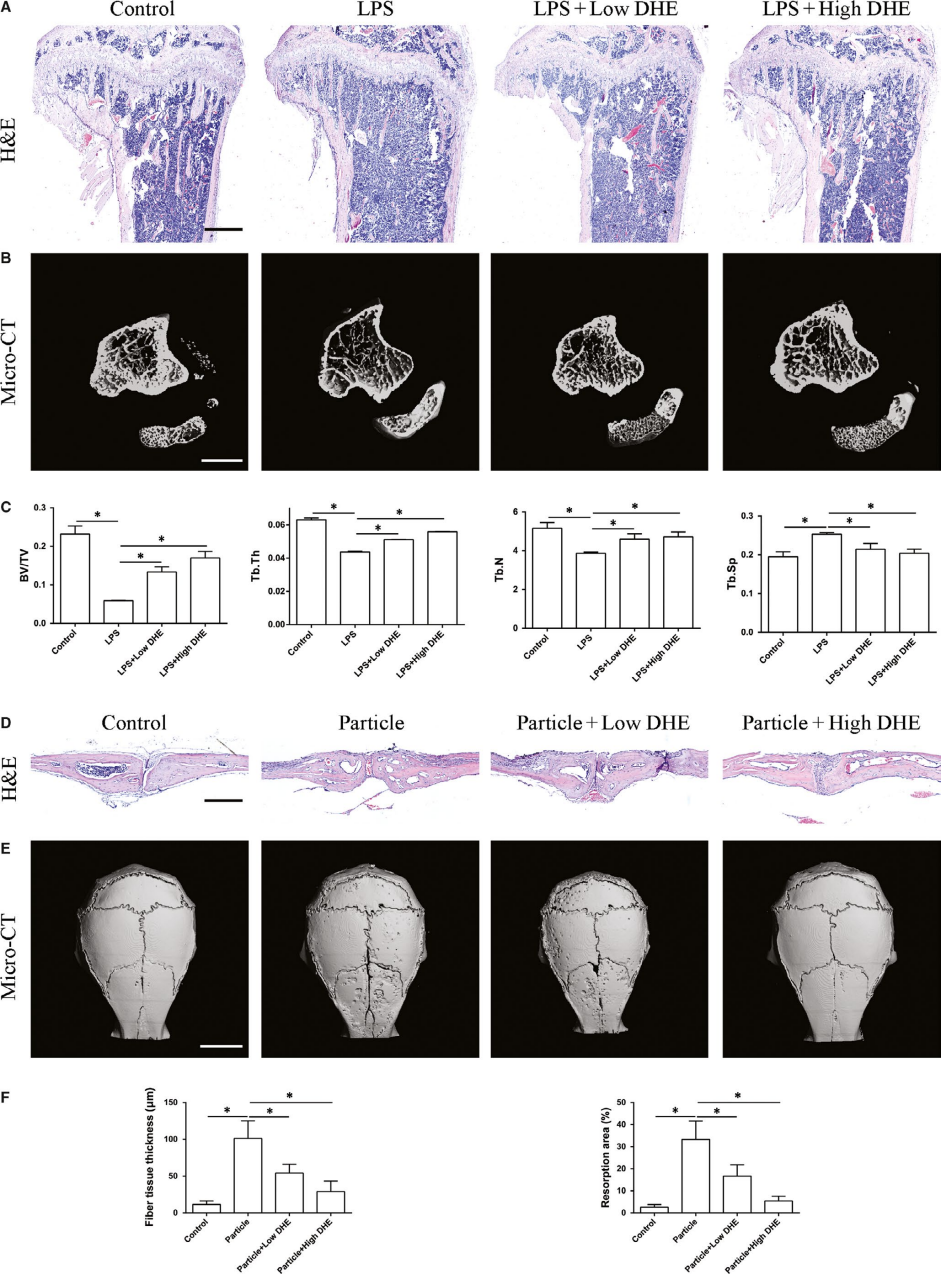
Dehydrocostus lactone (DHE) mitigated lipopolysaccharide (LPS)‐induced bone loss and particle‐induced calvarial osteolysis in vivo. A, Haematoxylin and eosin staining of representative sections of the proximal tibia in each group. Scale bar = 500 μm. B, Representative three‐dimensional micro‐CT reconstruction images of each group. Scale bar = 2 mm. C, Micro‐CT analyses of bone volume/tissue volume, trabecular number, trabecular thickness and trabecular separation in the region of interest. D, Haematoxylin and eosin staining of representative sections of the calvaria in each group. Scale bar = 500 μm. E, Representative three‐dimensional micro‐CT reconstruction images of each group. Scale bar = 3 mm. F, Quantification of fibre tissue thickness above the resorption area and resorption area of each group. Data are expressed as means ± SD. *P < 0.05 among groups

To characterize the antiresorptive property of DHE in vivo, models of LPS‐induced bone loss and particle‐induced calvarial osteolysis were adopted. Mice that received intraperitoneal LPS showed a significant bone volume loss at the secondary spongiosa of the tibia, while mice that received LPS + DHE showed a dose‐dependent increase in bone volume at this region (Figure 6A‐C). Three‐dimensional micro‐CT confirmed massive trabecular bone loss in LPS‐treated mice compared with control mice (Figure 6B). Consistent with the histological data, a morphometric study by micro‐CT indicated significant reductions in BV/TV, Tb.Th, and Tb.N, and a remarkable increase in Tb.Sp after LPS administration. Treatment with DHE partially prevented inflammatory bone volume loss in a dose‐dependent manner. What's more, protective effects of DHE were also observed in a model of particle‐induced calvarial osteolysis. The resorption area of calvaria was significantly reduced in DHE treated groups (Figure 6D‐F). Together, these results confirmed the protective effects of DHE on osteoclast‐related bone loss in vivo.

## 4 DISCUSSION

DHE is a naturally occurring sesquiterpene lactone reported to have many biological effects, including anticancer, bactericidal and antioxidative stress properties.^9^, ^14^, ^15^ However, there has been no report examining the effects of DHE on osteoclasts. In this study, we investigated its effects on RANKL‐induced osteoclast formation and function, and determined the underlying mechanism in vitro. We also examined its effects on LPS‐induced bone loss and particle‐induced calvarial osteolysis in vivo.

NFATc1 is a central regulator of the expression of marker genes, such as DC‐STAMP, cathepsin K, CTR, TRAP, MMP‐9 and V‐ATPase a3 in the differentiation of precursors into functional osteoclasts. In this study, we showed that treatment with DHE suppressed the mRNA expression of osteoclast marker genes, including those encoding NFATc1, DC‐STAMP, cathepsin K, CTR, TRAP, MMP‐9, V‐ATPase a3, and OSCAR. An in vitro study also confirmed the inhibitory effects of DHE on F‐actin ring formation, which is essential for bone resorption and osteoclast adhesion to bone.^16^

RAW264.7 cell line and BMMs are both macrophages and can differentiate into mature bone‐resorbing osteoclasts. The optimal cell seeding density, RANKL stimulating time point for differentiation of RAW264.7 cells into osteoclasts has been well defined by Song et al.^17^ But we hold the idea that RAW264.7 cell line may have limited advantage over BMMs in osteoclast differentiation and bone metabolism research other than its easy access, as in vitro study using BMMs would better mimic the in vivo situation. The proliferation of BMMs and their differentiation into osteoclasts are induced by M‐CSF and RANKL. RANKL‐induced signalling is critical to the formation of mature osteoclasts. The binding of RANKL and its receptor, RANK, leads to the recruitment of TRAF6.^5^ The MAPK, NF‐κB and PI3K/Akt pathways are the most well‐defined pathways involved in osteoclast differentiation, and their importance has been reported in many studies.^18^, ^19^ TRAF6 is recruited after the binding of RANKL and RANK, and it eventually forms a complex with TAK1 and TAK1‐binding protein 2.^20^ The complex activates TAK1, leads to the stimulation of NF‐κB and AP‐1 via of IKK and p38 phosphorylation, respectively.^21^ The current results indicate that DHE inhibited the phosphorylation of NF‐κB signalling, and attenuated the expression of c‐Src, cathepsin K and NFATc1. However, DHE had little effect on the RANKL‐induced phosphorylation of MAPK pathway.

The key role of osteoclasts in inflammatory bone loss makes it attractive as a potential therapeutic agent. Lipopolysaccharide is a membrane component of Gram‐negative bacteria and has been used as a potent inducer of osteolytic bone loss.^22^ Studies have suggested that microbial‐associated molecular patterns can adhere to generate wear debris in arthroplasty cases and lead to substantial inflammation 23; among them, LPS is the best known. It is capable of promoting the secretion of proinflammatory cytokines 24 and inducing osteoclast formation and osteoclastic bone erosion 25; this was reflected in the model of LPS‐induced inflammatory bone loss in our study. At the same time, bone histomorphometry indicated that DHE partially attenuated LPS‐induced inflammatory bone loss in vivo. What's more, the anti‐resorption activity of DHE was further consolidated by the particle‐induced calvarial osteolysis model. Our results are consistent with the possible use of DHE as a therapeutic agent for the treatment of osteoclast‐related bone loss.

## 5 CONCLUSION

Our findings demonstrate that DHE attenuated RANKL‐induced osteoclast formation and inhibited F‐actin ring formation and bone resorption in vitro. Moreover, DHE partially restored LPS‐induced bone loss in cancellous bone and particle‐induced calvarial osteolysis in vivo. Its mechanism involved suppression of the expression of NFATc1 and other marker genes during osteoclast formation by targeting the activation of NF‐κB signalling. These data suggest that DHE could be used for the treatment of osteoclast‐related skeletal disorders.

## CONFLICT OF INTEREST

The authors confirm that there are no conflicts of interest.

## AUTHORS’ CONTRIBUTION

BHu, FFW and ZLS performed the research, BHu and XZ analysed the data, BHe performed part of the *in vivo* study, BHu, HBW and SGY designed the research study and wrote the paper.
